# Supporting mental health through meditation: Perspectives from older adults with chronic diseases

**DOI:** 10.1192/j.eurpsy.2026.10662

**Published:** 2026-07-31

**Authors:** S. von Humboldt, I. Leal

**Affiliations:** William James Center for Research, ISPA – Instituto Universitário, Lisbon, Portugal

## Abstract

**Introduction:**

Meditation may serve as a protective factor for mental well-being in older adults, helping them cope with the ongoing challenges of chronic illness.

**Objectives:**

The objectives are to: 1) explore the emotional experiences of meditation among older adults living with chronic diseases, and 2) examine the impact of meditation on their mental health.

**Methods:**

The study involved 426 participants aged 65 to 89 years (*M* = 74.7; *SD* = 4.76) from Portuguese, English, and Brazilian backgrounds. Data were collected through in-depth, semi-structured interviews and analyzed using content analysis to identify key themes from participants’ experiences.

**Results:**

For the first objective, six themes emerged: Navigating illness and physical decline (90.4%), Seeking meaning and purpose in life (86.6%), Strengthening self-identity (72.5%), Experiencing reduced dependence and pain (70.2%); Cultivating hopefulness (64.8%); and Prioritizing effective disease management (62.0%). For the second objective, three themes were identified: Reduced emotional burden (83.8%), Decrease in depressive symptoms (78.6%), and Fewer emotional outbursts (52.1%). Portuguese participants emphasized navigating the challenges of illness and physical decline, whereas Brazilian participants placed greater importance on seeking life’s purpose. In contrast, English participants highlighted a stronger connection between meditation and strengthening their self-identity.

**Conclusions:**

The study reveals meditation’s significant impact on enhancing both physical and mental health among older adults living with chronic illnesses, demonstrating its value as a multifaceted support strategy in later years.

**Keywords:**

Chronic conditions; meditation; mental health; older adults; qualitative study.

**Disclosure of Interest:**

None Declared

